# Is Maintaining Thyroid-Stimulating Hormone Effective in Patients Undergoing Thyroid Lobectomy for Low-Risk Differentiated Thyroid Cancer? A Systematic Review and Meta-Analysis

**DOI:** 10.3390/cancers14061470

**Published:** 2022-03-13

**Authors:** Ho-Ryun Won, Eonju Jeon, Jae Won Chang, Yea Eun Kang, Kunho Song, Sun Wook Kim, Dong Mee Lim, Tae Kwun Ha, Ki-Wook Chung, Hyo-Jeong Kim, Young Joo Park, Bon Seok Koo

**Affiliations:** 1 Department of Otorhinolaryngology-Head and Neck Surgery, Chungnam National University College of Medicine, Daejeon 35015, Korea; hryun83@cnuh.co.kr (H.-R.W.); strive1005@cnuh.co.kr (J.W.C.); 2 Department of Otorhinolaryngology-Head and Neck Surgery, Chungnam National University Sejong Hospital, Sejong 30099, Korea; 3 Department of Internal Medicine, Daegu Catholic University School of Medicine, Daegu 42472, Korea; 0414mk@hanmail.net; 4 Department of Otorhinolaryngology-Head and Neck Surgery, Chungnam National University Hospital, Daejeon 35015, Korea; songkh87@cnuh.co.kr; 5 Division of Endocrinology and Metabolism, Departments of Internal Medicine, Chungnam National University Hospital, Daejeon 35015, Korea; yeeuni220@cnuh.co.kr; 6 Division of Endocrinology and Metabolism, Department of Medicine, Sungkyunkwan University School of Medicine, Seoul 16419, Korea; swkimmd@skku.edu; 7 Department of Internal Medicine, Konyang University Hospital, Daejeon 35365, Korea; mdldm@daum.net; 8 Department of Surgery, Inje University Busan Paik Hospital, Busan 47392, Korea; hasus@hanmail.net; 9 Department of Surgery, Ulsan University College of Medicine, Seoul 05505, Korea; surgeonckw@amc.seoul.kr; 10 Department of Internal Medicine, Eulji University College of Medicine, Daejeon 34824, Korea; kimhj@eulji.ac.kr; 11 Department of Internal Medicine, Seoul National University College of Medicine, Seoul 03080, Korea

**Keywords:** thyroid cancer, differentiated thyroid cancer, thyroid-stimulating hormone, thyroid lobectomy, recurrence rate

## Abstract

**Simple Summary:**

In order to reduce the recurrence rate after surgical treatment of differentiated thyroid cancer (DTC), suppression of thyroid-stimulating hormone (TSH) or maintenance of a certain level after surgery are important. However, the effectiveness of TSH maintenance in the mid to lower reference range (0.5–2 mU/L) in patients undergoing thyroid lobectomy for low-risk DTC is uncertain. In this systematic review and meta-analysis, we compared and analyzed the recurrence rate according to whether TSH maintenance was performed in patients who underwent thyroid lobectomy for low-risk DTC. There was no difference in the recurrence rate with or without TSH control. Therefore, the recommendation to maintain TSH to reduce the recurrence rate after thyroid lobectomy is still controversial.

**Abstract:**

There is no clear evidence that post-operative maintenance of thyroid-stimulating hormone (TSH) in the mid to lower reference range (0.5–2 mU/L) improves prognosis in patients undergoing thyroid lobectomy for low-risk differentiated thyroid cancer (DTC). The purpose of this systematic review and meta-analysis was to compare and analyze the recurrence rate according to whether the serum TSH level was maintained below 2 mU/L in patients who underwent thyroid lobectomy for low-risk DTC. Clinical data and outcomes were collected from MEDLINE, Embase, and the Cochrane Database of Systematic Reviews. The inclusion criteria were related studies on TSH maintenance or serum TSH concentration after surgery for DTC. Seven observational studies with a total of 3974 patients were included in this study. In the patients who received TSH maintenance less than 2 mU/L, the recurrence rate during the follow-up period was 2.3%. A subgroup analysis of five studies showed that the odds ratio for recurrence in patients who received TSH maintenance was 1.45 (*p*-value = 0.45) compared to patients who did not receive TSH maintenance. In conclusion, the evidence for the effectiveness of post-operative TSH maintenance less than 2 mU/L in patients undergoing thyroid lobectomy for low-risk DTC is insufficient.

## 1. Introduction

Thyroid cancer is the most common endocrine tumor and the most common malignant tumor in the head and neck region. In 2020, there were approximately 580,000 cases worldwide, and thyroid cancer ranked in ninth place (3%) among all cancers in terms of incidence [1]. Differentiated thyroid cancer (DTC), a category that includes papillary thyroid carcinoma, follicular thyroid carcinoma, and Hürthle cell thyroid carcinoma, accounts for 86% of all thyroid cancers [2].

Characteristically, DTC has a good prognosis, with a 5-year disease-specific mortality rate of less than 2% [3,4]. The mortality rate is low because the underlying physiological factors of DTC are indolent. However, persistent structural disease or recurrence is found in approximately 30% of patients [5]. Therefore, the 2015 American Thyroid Association (ATA) guidelines classified the risk of structural disease recurrence into three levels (low, intermediate, and high) according to the histopathological factors identified after initial surgical treatment and recommended additional treatment after initial therapy [6].

Traditionally, prophylactic cervical lymph node dissection, adjuvant radio-iodine ablation, and thyroid-stimulating hormone (TSH) suppression by levothyroxine have been suggested as treatment methods to reduce the recurrence rate of DTC [7,8]. In particular, a retrospective study reporting that TSH suppression treatment reduced the recurrence rate was published in 1977 [9]. Since then, various studies investigating the effect of TSH suppression therapy on reducing the recurrence rate have been reported, based on which it has been considered a standard treatment [10,11]. However, it has also been reported that excessive TSH suppression causes several side effects (e.g., osteoporosis, and cardiovascular disease) [12].

In recent years, the diagnosis rate of low-risk DTC patients has increased due to the increasing frequency of early screening and advances in diagnostic devices [13]. In light of a study reporting that the therapeutic effect of total thyroidectomy was not higher than that of thyroid lobectomy in low-risk DTC patients [14,15,16], thyroid lobectomy was added as a treatment option in the 2015 ATA guidelines [6]. For this reason, the number of low-risk DTC patients and the number of patients undergoing thyroid lobectomy have been increasing [13,17]. The 2015 ATA guidelines recommend initial goals of post-treatment serum TSH levels even in patients with low-risk DTC (low risk DTC and lobectomy patients: 0.5–2 mU/L) [6]. However, there is a low level of evidence for the therapeutic effect of TSH maintenance in low-risk DTC patients [6,18], especially those who have undergone thyroid lobectomy. Therefore, substantial uncertainty remains regarding this issue.

This study analyzed studies reporting the recurrence rate of patients who received TSH maintenance less than 2 mU/L in patients who underwent thyroid lobectomy for low-risk DTC. In addition, we analyzed studies reporting odds ratios in recurrence rates with and without TSH maintenance. Based on these analyses, the purpose of this systematic review and meta-analysis was to evaluate the effectiveness of TSH maintenance less than 2 mU/L in patients treated with thyroid lobectomy for low-risk DTC.

## 2. Materials and Methods

### 2.1. Search Strategy

The overall analysis process was performed based on the guidelines recommended by the Preferred Reporting Items for Systematic Reviews and Meta-Analyses (PRISMA) [19]. This Systematic Review was registered in PROPERO and the registration number is CRD42021253377. Three databases (MEDLINE, Embase, and the Cochrane Database of Systematic Reviews) were used to search for related literature, and studies published in the period from 1974 to December 2021 were targeted. We expanded the selection of synonyms for search terms related to the topic and established a search strategy using all those terms. After checking the controlled vocabulary related to the search terms in MeSH and EMTREE, all related terms were included. Additionally, we included literature in which all relevant terms were used as text words (Supplementary Data S1). There were no restrictions on language, study type, study species, sex, and age when establishing the search strategy. Grey literature and abstracts were also included.

### 2.2. Eligibility Criteria

**Figure 1:**
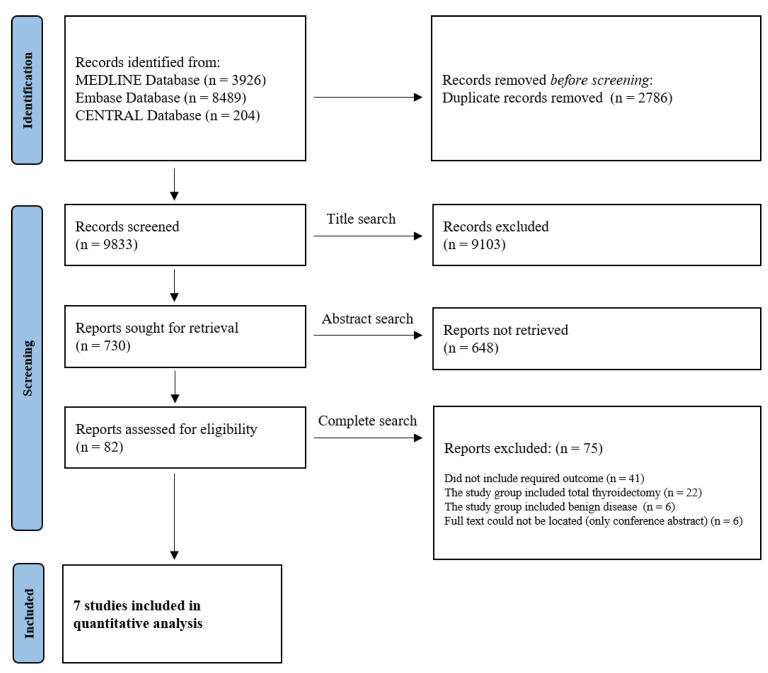
Flow diagram showing the selection of studies for inclusion in the systematic review and meta-analysis.

After applying the search strategy in three databases, a total of 9833 documents were identified when duplicate documents were excluded. The review process was conducted in three stages by two independent reviewers (H.R. Won and E. Jeon). In the first step, 730 relevant documents were identified through title review. Next, a full-text review was conducted for 82 studies identified through abstract review. In the final review stage, documents that met the following conditions were excluded: (1) the study population was not adults (18 years of age or older); (2) the required outcome and exposure were not included; (3) no full text was available (only abstracts). Finally, a total of seven studies were selected for the systematic review and meta-analysis (Figure 1). All steps were independently performed by each reviewer, and any discrepancies were resolved by discussion and consensus between the two reviewers.

### 2.3. Data Extraction

Data consistent with the purpose of this study were extracted by one reviewer (H.R. Won). The extracted data included: (1) the recurrence rate during follow-up in patients who underwent thyroid lobectomy after the diagnosis of DTC; and (2) serum TSH levels and related clinical outcomes. In addition, all clinical information that could affect clinical outcomes and data on treatment progress was also extracted.

### 2.4. Risk of Bias Analysis

**Figure 2:**
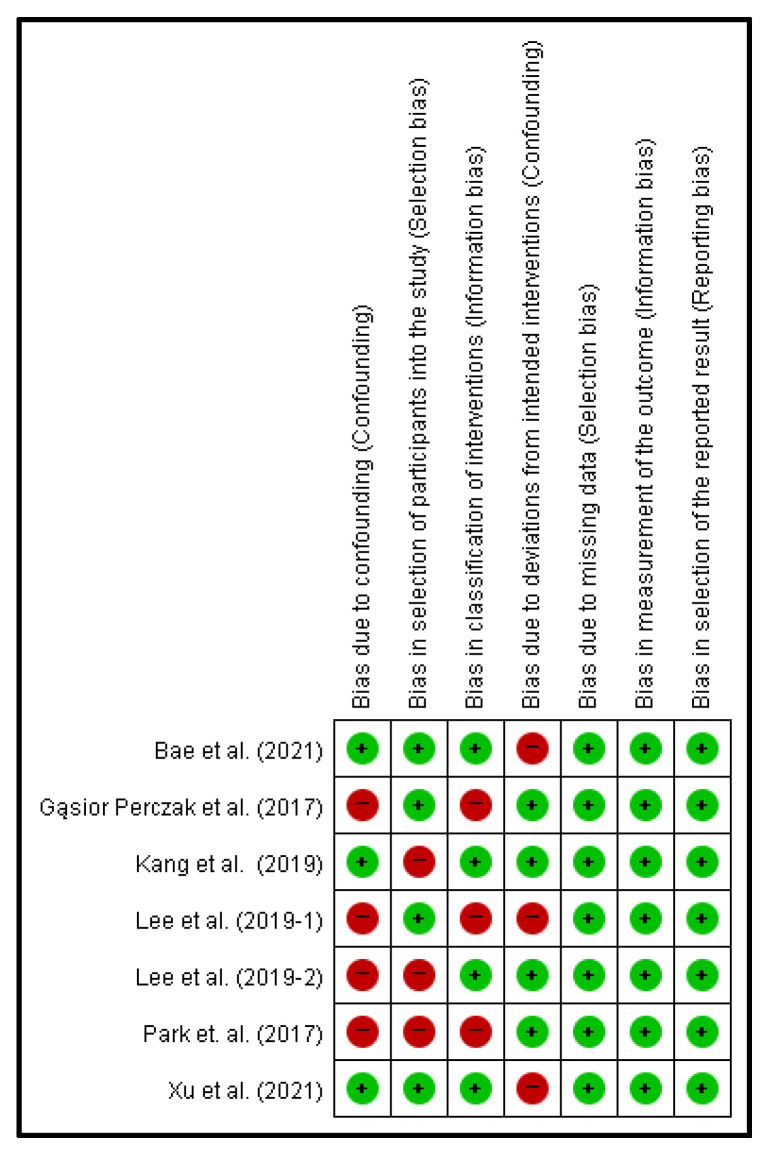
Risk of bias summary using ROBINS-I tool. (Red circle: high risk, green circle: low risk).

To evaluate the quality of non-randomized experimental studies, the Risk of Bias in Non-Randomized Studies of Interventions (ROBINS-I) tool was used [20]. Six items were evaluated and measured as low risk, high risk, and unclear risk. The results of the assessment of the risk of bias of the selected studies are shown in Figure 2. After two reviewers independently evaluated each item, items about which the reviewers disagreed were reanalyzed. The evaluation results were summarized using Review Manager 5.4 (Cochrane, London, UK).

### 2.5. Quantitative Data Analysis

After thyroid lobectomy for low-risk DTC, recurrence during the follow-up period of patients who underwent TSH maintenance less than 2 mU/L was extracted as the event rate. Both studies in which TSH maintenance was performed under the principle of maintaining the serum TSH concentration below 2 mU/L and those in which the average actual serum TSH concentration was maintained below 2 mU/L were included. Confidence intervals (CIs) were calculated using the rate of events from each study in the Comprehensive Meta-Analysis program version 3 (Biostat, Inc., Englewood, NJ, USA). Studies in which recurrence rates were compared and analyzed according to whether TSH maintenance less than 2 mU/L was performed were designated as a subgroup, and an additional meta-analysis was performed. Comprehensive Meta-Analysis program version 3 was used for this analysis, and the results were expressed as the odds ratio. All meta-analyses were based on 95% CIs, and a random-effects model was used.

### 2.6. Assessment of Study Heterogeneity

In performing meta-analysis, a random-effects model was applied assuming that there is a difference between the target study groups. In a meta-analysis of seven studies, the I^2^ value was 87.43%, showing high heterogeneity. A subgroup meta-analysis of five studies showed moderate heterogeneity with an I^2^ value of 61.54%.

## 3. Results

### 3.1. Study Selection

Seven studies were selected and analyzed after the exclusion of studies that satisfied the exclusion criteria through a three-stage review process by two independent reviewers (Figure 1) [21,22,23,24,25,26,27]. A list of studies included in the analysis is summarized in Table 1, along with related information, required results, and data.

### 3.2. Recurrence Rate in Patients Who Received TSH Maintenance Less Than 2 mU/L after Thyroid Lobectomy for Low-Risk DTC

We extracted the recurrence rates reported in studies that received TSH maintenance in the mid to lower reference range (0.5–2 mU/L) after thyroid lobectomy according to the recommendation presented in the 2015 ATA guidelines [6]. In addition, since the standard for TSH maintenance suggested in the 2015 ATA guidelines is a serum TSH concentration less than 2 mU/L, data from studies reporting the recurrence rate in patients maintained below this concentration were also combined and analyzed. The mean follow-up period ranged from a minimum of 60 months to a maximum of 103.2 months. A meta-analysis using a random-effects model was performed, and the results are shown in Table 2. The recurrence rate for patients with TSH maintenance (serum TSH < 2 mU/L) after thyroid lobectomy was 2.3% (95% CI 1.0–5.2%).

### 3.3. Association between TSH Maintenance Less Than 2 mU/L and the Recurrence Rate in Patients with Thyroid Lobectomy for Low-Risk DTC

Studies that analyzed the recurrence rate according to whether TSH maintenance in the mid to lower reference range was performed according to the 2015 ATA guidelines, as well as those that analyzed the recurrence rate based on the standard of a serum TSH level of 2 mU/L, were set as a subgroup that included five studies [21,22,24,26,27]. When TSH maintenance was performed, the odds ratio of recurrence was 1.45 (95% CI 0.58–3.61) compared to the patients in whom TSH maintenance less than 2 mU/L was not performed (Table 3).

### 3.4. Clinical Outcomes Related to TSH Levels after Thyroid Lobectomy for Low-Risk DTC

TSH levels and related clinical outcomes reported from the seven studies included in the review are summarized in Table 4. In the study of Bae et al., there was a decreased tendency to increase TSH levels by >2 mU/L without T4 supplementation over time after thyroid lobectomy. In addition, it was reported that the postoperative TSH level was significantly more likely to increase to 2 mU/L or higher if the TSH level was 2 mU/L or higher before surgery [21]. Xu et al. reported that there was no significant difference between the 10-year recurrent-free survival rate and the disease-free survival (DFS) rate according to the postoperative TSH level [22]. Kang et al. reported that, if TSH maintenance in the mid to lower reference range was performed for one year in patients who underwent thyroid lobectomy, the probability that the serum TSH level would increase to 10 mU/L or higher was significantly decreased [24]. However, they reported that serum TSH level was not an independent risk factor for recurrence [24]. Park et al. reported that there was no significant relationship between TSH maintenance in the mid to lower reference range and DFS (*p* = 0.63). Additionally, they reported no significant difference when comparing DFS based on a serum TSH concentration of 2 mU/L (*p* = 0.85) [27]. A meta-analysis of clinical outcomes related to serum TSH levels was not possible due to the different statistical methods used in each study.

## 4. Discussion

Thyroid cancer is the most common endocrine cancer, ranked in ninth place (3%) among all cancers worldwide as of 2020 [1]. Especially in South Korea, the incidence rate has been high for several years. According to the South Korean national cancer statistics from 2018, it ranked in second place (19.1%) among malignancies in women after breast cancer [28]. DTC, which accounts for more than 86% of thyroid cancers [2], has a relatively good prognosis compared to other carcinomas. According to the eighth edition American Joint Committee on Cancer staging manual, the 10-year disease-specific survival is 99.8% for stage I and 88.3% for stage II [29]. In addition, patients diagnosed with stage I or II account for 95% of all DTC patients [29]. While the prognosis of DTC is relatively good, the recurrence rate is still non-negligible. Therefore, for the systematic management and treatment of recurrence, the 2015 ATA guidelines proposed a risk stratification system that divides the risk of recurrence into three levels (low, intermediate, and high) [6]. A recent study reported that, in patients who underwent radio-iodine ablation after total thyroidectomy with DTC, persistent structural disease or recurrence occurred in 3% of those at low risk, 21% of those at intermediate risk, and 68% of those at high risk [5]. As a result, the importance of systematic management and adjuvant therapy is being emphasized to reduce the recurrence rate [30].

TSH suppression therapy is a method traditionally used to reduce the recurrence rate of DTC. TSH receptors exist in the cell membrane of DTC, and their stimulation promotes cell growth by increasing the expression of several thyroid-related proteins, such as thyroglobulin [31]. The main mechanism of TSH suppression therapy is to reduce cell proliferation by inhibiting the TSH receptor through levothyroxine [12]. In 1977, Mazzaferri et al. published a study finding that the recurrence rate was significantly reduced after TSH suppression [9,12]. Since then, this effect of TSH suppression has been reported in several studies [10,32,33,34], and a meta-analysis by McGriff et al. reported that the risk of major adverse clinical events, such as mortality and recurrence, was significantly reduced after TSH suppression [35]. Additionally, Hovens et al. reported that adverse effects, such as the recurrence of DTC or thyroid cancer-related death, became evident when the serum TSH concentration was 2 mU/L or higher, and strongly advised that the TSH concentration should be kept below 0.1 mU/L in the high-risk group and those with recurrent tumors [36].

Based on these results, recommendations for TSH suppression after initial treatment of DTC were published in the 2015 ATA guidelines [6]. However, most of the studies were conducted among patients with high-risk DTC; therefore, the initial goal of post-treatment serum TSH level for patients at intermediate or low risk, especially after thyroid lobectomy, is still weak [6]. A recent study even reported that TSH suppression prior to the initial post-treatment evaluation in low- to intermediate-risk DTC patients had no significant effect on structural recurrence [37]. In addition, in the randomized controlled trial conducted by Sugitani et al., it was recommended to avoid TSH suppression in low-risk DTC patients considering its side effects [12]. As increasingly many cases of low-risk DTC are diagnosed and thyroid lobectomy is frequently performed as the initial treatment [13,17,38], research is needed to clarify the rationale for setting the TSH goal after thyroid lobectomy. Therefore, we conducted this systematic review and meta-analysis of studies and literature related to TSH maintenance after thyroid lobectomy.

When we analyzed the data extracted from seven studies, the recurrence rate was found to be 2.3% when TSH maintenance less than 2 mU/L was performed after thyroid lobectomy for low-risk DTC. Several previous studies on the recurrence rate after thyroid lobectomy for low-risk DTC reported rates of 1.7% to 8.2% [16,26,39,40], and a recently published meta-analysis reported that recurrence occurred in up to 9.0% of patients [41]. Although the recurrence rate of 2.3% found in this study is relatively low compared to the recurrence rates reported in previous studies, it was found to be within the range of previous reports. Furthermore, an additional subgroup analysis of five studies showed that the odds ratio of recurrence was 1.45 (*p* = 0.43) when TSH maintenance was received compared to patients who did not receive TSH maintenance less than 2 mU/L. While not statistically significant, there was a tendency for the recurrence ratio to be higher in patients who received TSH maintenance.

Some studies reported interesting results, although they were not included in the analysis of this study due to the inconsistency of the data. Matsuzu et al. reported that only 6.5% of patients who underwent TSH maintenance after thyroid lobectomy experienced recurrence over 25 years, which is similar to the recurrence rate in previous studies. However, in that study, specific criteria for TSH maintenance and results for the serum TSH concentration were omitted [16]. Additionally, Ann et al. analyzed the prognosis of DTC according to thyroid hormone supplementation after thyroid lobectomy. Although post-operative hypothyroidism was not a risk factor for recurrence of DTC, levothyroxine supplementation that did not induce TSH suppression reduced the recurrence rate [42]. In contrast, another study (Park et al.) reported that DFS was significantly higher in the group with low serum TSH levels of 1.85 mU/L (*p* = 0.012) [43]. In addition, recently, Xiang et al. reported that DFS was significantly worse when TSH level was higher than 2.615 after surgery, which was more obvious in patients with the BRAF mutation [44].

This systematic review and meta-analysis have several limitations. Most importantly, there were no randomized controlled trials, and the number of observational studies that could be analyzed was small. Only studies that clearly stated that TSH was maintained according to the recommendations of the 2015 ATA guidelines were selected. We also selected studies that specified recurrence as a study outcome. As a result, there were only seven publications consistent with the inclusion criteria of this study. Therefore, the level of evidence furnished by this study is low.

Another limitation is that studies in which the serum TSH concentration was maintained below 2 mU/L and studies that attempted to keep it below 2 mU/L were combined for analysis. Serum TSH concentrations can change frequently. In the latter studies, even if an attempt was made to keep the serum concentration of TSH below 2 mU/L according to the criteria, there is a possibility that it may not actually have been maintained below 2 mU/L. Two studies included in this analysis used the median serum TSH concentration [22,26]. More generally, even if the goal was to maintain the TSH concentration below 2 mU/L, it cannot be concluded that the TSH concentration showed no variation; this issue could impair the accuracy of the results of this study. Furthermore, the heterogeneity evaluation showed an I^2^ value of 87.43% in the recurrence rate analysis, which is a relatively high value. In the subgroup analysis, the recurrence rate according to the maintenance of serum TSH showed contradictory results depending on the study. As a result, in the subgroup analysis, the I^2^ value was 61.54%, showing moderate heterogeneity. In particular, the results of Bae et al. were included in the meta-analysis due to overlapping CIs. However, since it showed a notable difference compared to other studies, it is thought to have increased the heterogeneity of the meta-analysis.

Lastly, inconsistencies in the characteristics of the study subjects, such as whether central neck dissection was performed, and the length of follow-up and pathological variants that may affect recurrence rate, also weaken the evidence for the outcome. In light of the findings of this study, there is a clear need for large-scale randomized controlled trials on the effectiveness of TSH maintenance after thyroid lobectomy for low-risk DTC patients. In addition, in future studies, an analysis of the side effects accompanying TSH maintenance less than 2 mU/L, along with the effectiveness of TSH maintenance, should be simultaneously performed.

## 5. Conclusions

In the present systematic review and meta-analysis, the following results were obtained. After thyroid lobectomy for low-risk DTC patients, when TSH control to maintain the serum TSH concentration below 2 mU/L was performed, the recurrence rate was 2.3% during the follow-up period. The application of TSH maintenance less than 2 mU/L did not significantly affect the odds ratio for recurrence. For this reason, based on the studies conducted to date, the evidence for consistent application of TSH maintenance less than 2 mU/L in patients undergoing thyroid lobectomy is weak and insufficient. Therefore, the application of TSH maintenance less than 2 mU/L in patients who have undergone thyroid lobectomy for low-risk DTC should be carefully determined by considering the possible side effects as well as ensuring an accurate clinical assessment of each patient.

## Figures and Tables

**Table 1:** Studies included in the systematic review and meta-analysis.

Study	Study Design	Participant (n)		Age Mean (SD)	Gender (Male/ Female) (%)	ATA Risk	CND	Pathology	Follow Up Mean, Month (SD)	Outcome (Incidence of Recurrence) (%)	Comments
		No TSH Maintenance (≥2 mU/L)	TSH Maintenance (<2 mU/L)								
Bae et al. [21]	Retrospective analysis	134	235	49 (NR)	89 (24.1%)/280 (75.9%)	low	Performed	PTC-NR	72 (NR)	4/134 (2.99%): 0/235 (0%)	Patients were indicated to receive T4 supplementation according to the 2015 ATA guidelines
Xu et al. [22]	Retrospective analysis	189	757	42 (NR) (All participant)	547 (23.8%)/1750 (76.2%) (All participant)	low	Performed depending on the surgeon’s preference	PTC-CV, FV	70 (NR)	10/189 (5.29%): 65/757 (8.59%)	TSH value is derived by calculating the mean value for course of follow-up.
Gąsior Perczak et al. [23]	Retrospective analysis	NA	102	49.8 (13.6)	8 (7.8%)/ 94 (92.2%)	low	Not performed	PTC-CV, FV	60 (40.8)	2/102 (1.96%)	TSH levels were within the recommended range for the patients (0.5–2 mU/L)
Kang et al. [24]	Retrospective analysis	100	100	42.79 (9.60): 45.43 (8.90)	13 (13%)/ 87 (87%): 14 (14%)/ 86 (86%)	low	Performed depending on the surgeon’s preference	PTC-NR	More than 60 (NR)	2/100 (2%): 5/100 (5%)	Check TSH suppression by administering the same dose of LT4. TSH suppression performed only 1 year after surgery.
Lee et al. [25]	Retrospective/prospective analysis	NA	363	52.21 (9.88)	67 (18.5%)/296 (81.5%)	low	Performed	PTC-NR	67 (NR)	1/363 (0.3%)	No description of the method in TSH maintenance. ATA 2015 guidelines are presented in the introduction.
Lee et al. [26]	Retrospective analysis	863	665	47 (10) (All participant)	177 (12%)/1351 (88%) (All participant)	low	Performed depending on the surgeon’s preference	PTC-CV, FV	67.2 (NR)	5/863 (0.58%): 16/665 (2.40%)	TSH value is derived by calculating the mean value for 5 years.
Park et al. [27]	Retrospective analysis (cohort study)	233	233	47.60 (10.26): 47.23 (10.00)	39 (16.7%)/ 194(83.3%): 31 (13.3%)/202 (86.7%)	low	Performed	PTC-CV, FV/ FTC	Median 103.2 (NR)	6/233 (2.58%): 4/233 (1.72%)	Groups are classified based on TSH maintenance.

TSH, thyroid stimulating hormone; SD, standard deviation; ATA, American Thyroid Association; CND, central neck dissection; NA, not applicable; PTC, papillary thyroid carcinoma; NR, not reported; LT4, levothyroxine; CV, classic variant; FV, follicular variant; FTC, follicular thyroid carcinoma.

**Table 2:** Recurrence rate in patients who received TSH maintenance less than 2 mU/L after thyroid lobectomy for low-risk differentiated thyroid cancer.

Study	Statistics for Each Study					Weight (%)	Event Rate and 95% CI
	Event Rate	Lower Limit	Upper Limit	Z-Value	*p*-Value		
Bae et al. [21]	0.002	0.000	0.033	−4.348	0.000	6.49	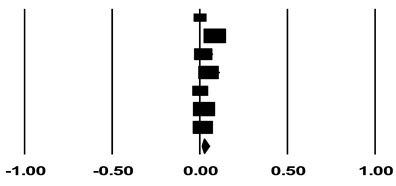
Xu et al. [22]	0.086	0.068	0.108	−18.232	0.000	19.66	
Gąsior Perczak et al. [23]	0.020	0.005	0.075	−5.478	0.000	13.07	
Kang et al. [24]	0.050	0.021	0.115	−6.417	0.000	16.41	
Lee et al. [25]	0.003	0.000	0.019	−5.884	0.000	9.79	
Lee et al. [26]	0.024	0.015	0.039	−14.632	0.000	18.75	
Park et al. [27]	0.017	0.006	0.045	−8.025	0.000	15.82	
**Random**	**0.023**	**0.010**	**0.052**	**−8.595**	**0.000**		
Heterogeneity: Tau^2^ = 0.962; df = 6 (*p* = 0.000); I^2^ = 87.433%							

CI, confidence interval.

**Table 3:** Association between TSH maintenance less than 2 mU/L and recurrence rate in patients with lobectomy for low-risk differentiated thyroid cancer.

Study	Statistics for Each Study					Weight (%)	Odds Ratio and 95% CI
	Odds Ratio	Lower Limit	Upper Limit	Z-Value	*p*-Value		
Bae et al. [21]	0.062	0.003	1.153	−1.865	0.062	7.64	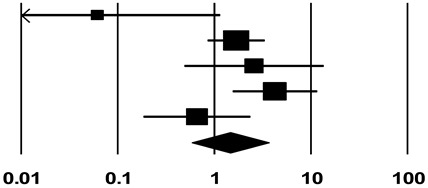
Xu et al. [22]	1.618	0.847	3.338	1.485	0.138	29.91	
Kang et al. [24]	2.579	0.488	13.617	1.116	0.264	16.38	
Lee et al. [26]	4.231	1.542	11.608	2.801	0.005	24.98	
Park et al. [27]	0.661	0.184	2.373	−0.635	0.525	21.09	
**Random**	**1.449**	**0.582**	**3.607**	**0.796**	**0.426**		
Heterogeneity: Tau^2^ = 0.602; df = 4 (*p* = 0.034); I^2^ = 61.538%							

CI, confidence interval.

**Table 4:** TSH levels reported in studies included in the systematic review and meta-analysis.

Study (Year)	Participant (n)		TSH Level (mU/L, Mean ± SD)	Clinical Outcome Related TSH Level	Comments
	No TSH Maintenance (≥2 mU/L)	TSH Maintenance (<2 mU/L)			
Bae et al. [21]	134	235	Serum TSH concentrations: proportions with TSH > 2 mU/L post-lobectomy 1 month: 77.0% 3–6 months: 82.3% 12 months: 66.7% 24 months: 59.9%	Preoperative TSH level (OR = 2.182, 95% CI, 1.301–3.659; *p* = 0.003) was the independent variable that predicted the need for TSH suppression.	
Xu et al. [22]	189	757	TSH level ≤ 0.5 (n = 254) TSH level 0.5–2 (n = 503) TSH level 2–4 (n = 135) TSH level > 4 (n = 54)	10-year RFS rate TSH level ≤ 0.5: 95.1% TSH level 0.5–2: 89.4% TSH level 2–4: 96.1% TSH level > 4: 91.2% Compare DFS and TSH level 0.5–2 and 2–4 *p* = 0.997 0.5–2 and >4 *p* = 0.487	
Gąsior Perczak et al. [23]	NA	102	Only report TSH levels in recurrence patients (1.86)	NR	
Kang et al. [24]	100	100	Postoperative TSH > 10 patient’s number: No TSH maintenance: 25 (25%) TSH maintenance: 13 (13%)	1-year TSH maintenance effect on postoperative TSH >10 *p* = 0.036	
Lee et al. [25]	NA	363	Failure to cessation of TSH maintenance (0.90 ± 0.82) (n = 170) Success to cessation of TSH maintenance (0.96 ± 0.98) (n = 193)	NR	
Lee et al. [26]	863	665	TSH level ≥ 2 (n = 863) TSH level < 0.5 (n = 115) TSH level 0.5–1.9 (n = 550) Mean TSH levels: NR	Hazard ratio (95%CI) according to RFS and mean TSH Univariate: <0.5: 0.49 (0.14–1.64) 0.5–1.9: Ref. 2.0–4.4: 0.39 (0.12–1.24) ≥4.5: 0.36 (0.04–2.88) Multivariate: <0.5: 0.44 (0.12–1.61) 0.5–1.9: Ref. 2.0–4.4: 0.35 (0.11–1.13) ≥4.5: 0.31 (0.03–2.58)	
Park et al. [27]	233	233	Mean TSH levels: NR	Compare DFS and TSH level (TSH level ≥2 and <2) *p* = 0.85	

TSH, thyroid stimulating hormone; SD, standard deviation; NA, not applicable; CI, confidence interval; NR, not reported; RFS, recurrent-free survival; DFS. disease-free survival.

## Data Availability

Some or all datasets generated during and/or analyzed during the current study are not publicly available but are available from the corresponding author on reasonable request.

## Supplementary Materials

The following are available online at https://www.mdpi.com/article/10.3390/cancers14061470/s1, Supplementary Data S1: Search strategy.
