# Supplementary material for: Is Maintaining Thyroid-Stimulating Hormone Effective in Patients Undergoing Thyroid Lobectomy for Low-Risk Differentiated Thyroid Cancer? A Systematic Review and Meta-Analysis
Source: Cancers (Basel). 2022 Mar 13;14(6):1470. doi: 10.3390/cancers14061470 (PMC8946503; doi:10.3390/cancers14061470)
Supplement: Supplementary file 1 [file cancers-14-01470-s001.zip › cancers-1589643-supplementary.pdf]

Search strategy

MEDLINE (1946~)

1. "thyroid neoplasms"[MeSH Terms] OR ("thyroid"[All Fields] AND "neoplasms"[All Fields]) OR "thyroid neoplasms"[All Fields] OR ("thyroid"[All Fields] AND "neoplasm"[All Fields]) OR "thyroid neoplasm"[All Fields] OR ("thyroid neoplasms"[MeSH Terms] OR ("thyroid"[All Fields] AND "neoplasms"[All Fields]) OR "thyroid neoplasms"[All Fields] OR ("thyroid"[All Fields] AND "cancer"[All Fields]) OR "thyroid cancer"[All Fields]) OR ("thyroid neoplasms"[MeSH Terms] OR ("thyroid"[All Fields] AND "neoplasms"[All Fields]) OR "thyroid neoplasms"[All Fields] OR ("thyroid"[All Fields] AND "carcinoma"[All Fields]) OR "thyroid carcinoma"[All Fields]) OR ("thyroid cancer, papillary"[MeSH Terms] OR ("thyroid"[All Fields] AND "cancer"[All Fields] AND "papillary"[All Fields]) OR "papillary thyroid cancer"[All Fields] OR ("papillary"[All Fields] AND "thyroid"[All Fields] AND "carcinoma"[All Fields]) OR "papillary thyroid carcinoma"[All Fields]) OR ("thyroid cancer, papillary"[MeSH Terms] OR ("thyroid"[All Fields] AND "cancer"[All Fields] AND "papillary"[All Fields]) OR "papillary thyroid cancer"[All Fields] OR ("papillary"[All Fields] AND "thyroid"[All Fields] AND "cancer"[All Fields])) OR (("cell differentiation"[MeSH Terms] OR ("cell"[All Fields] AND "differentiation"[All Fields]) OR "cell differentiation"[All Fields] OR "differentiated"[All Fields] OR "differentiation"[All Fields] OR "differential"[All Fields] OR "differentials"[All Fields] OR "differentiate"[All Fields] OR "differentiates"[All Fields] OR "differentiating"[All Fields] OR "differentiational"[All Fields] OR "differentiations"[All Fields] OR "differentiative"[All Fields]) AND ("thyroid neoplasms"[MeSH Terms] OR ("thyroid"[All Fields] AND "neoplasms"[All Fields]) OR "thyroid neoplasms"[All Fields] OR ("thyroid"[All Fields] AND "cancer"[All Fields]) OR "thyroid cancer"[All Fields])) OR (("cell differentiation"[MeSH Terms] OR ("cell"[All Fields] AND "differentiation"[All Fields]) OR "cell differentiation"[All Fields] OR "differentiated"[All Fields] OR "differentiation"[All Fields] OR "differential"[All Fields] OR "differentials"[All Fields] OR "differentiate"[All Fields] OR "differentiates"[All Fields] OR "differentiating"[All Fields] OR "differentiational"[All Fields] OR "differentiations"[All Fields] OR "differentiative"[All Fields]) AND ("thyroid neoplasms"[MeSH Terms] OR ("thyroid"[All Fields] AND "neoplasms"[All Fields]) OR "thyroid neoplasms"[All Fields] OR ("thyroid"[All Fields] AND "carcinoma"[All Fields]) OR "thyroid carcinoma"[All Fields])) OR ("thyroid neoplasms"[MeSH Terms] OR ("thyroid"[All Fields] AND "neoplasms"[All Fields]) OR "thyroid neoplasms"[All Fields] OR ("thyroid"[All Fields] AND "tumor"[All Fields]) OR "thyroid tumor"[All Fields])

94,374

2. "thyroidectomy"[MeSH Terms] OR "thyroidectomy"[All Fields] OR "thyroidectomies"[All Fields] OR ("hemithyroidectomies"[All Fields] OR "hemithyroidectomy"[All Fields]) OR (("thyroid gland"[MeSH Terms] OR ("thyroid"[All Fields] AND "gland"[All Fields]) OR "thyroid gland"[All Fields] OR "thyroid"[All Fields] OR "thyroid usp"[MeSH Terms] OR ("thyroid"[All Fields] AND "usp"[All Fields]) OR "thyroid usp"[All Fields] OR "thyroids"[All Fields] OR "thyroid s"[All Fields] OR "thyroidal"[All Fields] OR "thyroideal"[All Fields] OR "thyroidism"[All Fields] OR "thyroiditis"[MeSH Terms] OR "thyroiditis"[All Fields] OR "thyroiditides"[All Fields]) AND ("lobectomies"[All Fields] OR "lobectomy"[All Fields])) OR (("subtotal"[All Fields] OR "subtotally"[All Fields] OR "subtotals"[All Fields]) AND ("thyroidectomy"[MeSH Terms] OR "thyroidectomy"[All Fields] OR "thyroidectomies"[All Fields])) 32,777

3. "thyrotropin"[MeSH Terms] OR "thyrotropin"[All Fields] OR ("thyroid"[All Fields] AND "stimulating"[All Fields] AND "hormone"[All Fields]) OR "thyroid stimulating hormone"[All Fields] OR (("thyroid hormones"[MeSH Terms] OR ("thyroid"[All Fields] AND "hormones"[All Fields]) OR "thyroid hormones"[All Fields] OR ("thyroid"[All Fields] AND "hormone"[All Fields]) OR "thyroid hormone"[All Fields]) AND ("therapeutics"[MeSH Terms] OR "therapeutics"[All Fields] OR "therapies"[All Fields] OR "therapy"[MeSH Subheading] OR "therapy"[All Fields] OR "therapy s"[All Fields] OR "therapys"[All Fields])) OR ("thyroid hormones"[MeSH Terms] OR ("thyroid"[All Fields] AND "hormones"[All Fields]) OR "thyroid hormones"[All Fields] OR ("thyroid"[All Fields] AND "hormone"[All Fields]) OR "thyroid hormone"[All Fields]) OR ("thyroxine"[MeSH Terms] OR "thyroxine"[All Fields] OR "thyroxin"[All Fields] OR "thyroxines"[All Fields]) OR ("laevothyroxine"[All Fields] OR "thyroxine"[MeSH Terms] OR "thyroxine"[All Fields] OR "levothyroxine"[All Fields] OR "levothyroxin"[All Fields]) OR "TSH"[All Fields] OR ("TSH"[All Fields] AND ("suppress"[All Fields] OR "suppressed"[All Fields] OR "suppresser"[All Fields] OR "suppresses"[All Fields] OR "suppressibility"[All Fields] OR "suppressible"[All Fields] OR "suppressing"[All Fields] OR "suppression"[All Fields] OR "suppressions"[All Fields] OR "suppressive"[All Fields] OR "suppressives"[All Fields])) OR (("thyroid hormones"[MeSH Terms] OR ("thyroid"[All Fields] AND "hormones"[All Fields]) OR "thyroid hormones"[All Fields] OR ("thyroid"[All Fields] AND "hormone"[All Fields]) OR "thyroid hormone"[All Fields]) AND ("suppress"[All Fields] OR "suppressed"[All Fields] OR "suppresser"[All Fields] OR "suppresses"[All Fields] OR "suppressibility"[All Fields] OR "suppressible"[All Fields] OR "suppressing"[All Fields] OR "suppression"[All Fields] OR "suppressions"[All Fields] OR "suppressive"[All

Fields] OR "suppressives"[All Fields])) OR (("thyrotropin"[MeSH Terms] OR "thyrotropin"[All Fields] OR ("thyroid"[All Fields] AND "stimulating"[All Fields] AND "hormone"[All Fields]) OR "thyroid stimulating hormone"[All Fields]) AND ("suppress"[All Fields] OR "suppressed"[All Fields] OR "suppresser"[All Fields] OR "suppresses"[All Fields] OR "suppressibility"[All Fields] OR "suppressible"[All Fields] OR "suppressing"[All Fields] OR "suppression"[All Fields] OR "suppressions"[All Fields] OR "suppressive"[All Fields] OR "suppressives"[All Fields])) OR "T4"[All Fields] OR ("thyrotropin"[MeSH Terms] OR "thyrotropin"[All Fields] OR "thyrotropine"[All Fields] OR "thyrotropins"[All Fields]) 190,224

4. #1 AND #2 AND #3 3,926

EMBASE (1974~)

1. 'thyroid neoplasm'/exp OR 'thyroid neoplasm' OR (('thyroid'/exp OR thyroid) AND ('neoplasm'/exp OR neoplasm)) OR 'thyroid tumor'/exp OR 'thyroid tumor' OR (('thyroid'/exp OR thyroid) AND ('tumor'/exp OR tumor)) OR 'thyroid cancer'/exp OR 'thyroid cancer' OR (('thyroid'/exp OR thyroid) AND ('cancer'/exp OR cancer)) OR 'thyroid carcinoma'/exp OR 'thyroid carcinoma' OR (('thyroid'/exp OR thyroid) AND ('carcinoma'/exp OR carcinoma)) OR 'papillary thyroid carcinoma'/exp OR 'papillary thyroid carcinoma' OR (papillary AND ('thyroid'/exp OR thyroid) AND ('carcinoma'/exp OR carcinoma)) OR 'differentiated thyroid cancer'/exp OR 'differentiated thyroid cancer' OR (differentiated AND ('thyroid'/exp OR thyroid) AND ('cancer'/exp OR cancer)) OR 'differentiated thyroid carcinoma'/exp OR 'differentiated thyroid carcinoma' OR (differentiated AND ('thyroid'/exp OR thyroid) AND ('carcinoma'/exp OR carcinoma)) 148,053

2. 'thyroidectomy'/exp OR thyroidectomy OR 'hemithyroidectomy'/exp OR hemithyroidectomy OR 'thyroid lobectomy'/exp OR 'thyroid lobectomy' OR (('thyroid'/exp OR thyroid) AND ('lobectomy'/exp OR lobectomy)) OR 'subtotal thyroidectomy'/exp OR 'subtotal thyroidectomy' OR (subtotal AND ('thyroidectomy'/exp OR thyroidectomy)) 47,517

3. 'thyroid stimulating hormone'/exp OR 'thyroid stimulating hormone' OR (('thyroid'/exp OR thyroid) AND stimulating AND ('hormone'/exp OR hormone)) OR 'thyroid hormone therapy' OR (('thyroid'/exp OR thyroid) AND ('hormone'/exp OR hormone) AND ('therapy'/exp OR therapy)) OR 'thyroid hormone'/exp OR 'thyroid hormone' OR (('thyroid'/exp OR thyroid) AND ('hormone'/exp OR hormone)) OR 'thyroxine'/exp OR thyroxine OR 'levothyroxine'/exp OR levothyroxine OR 'tsh'/exp OR tsh OR 'tsh suppression' OR (('tsh'/exp OR tsh) AND

('suppression'/exp OR suppression)) OR 'thyroid hormone suppression' OR (('thyroid'/exp OR thyroid) AND ('hormone'/exp OR hormone) AND ('suppression'/exp OR suppression)) OR 'thyroid stimulating hormone suppression' OR (('thyroid'/exp OR thyroid) AND stimulating AND ('hormone'/exp OR hormone) AND ('suppression'/exp OR suppression)) OR t4 OR 'thyrotropin'/exp OR thyrotropin 237,385

4. #1 AND #2 AND #3 8,489

CHOCRANE RIBRARY

1. thyroid neoplasm (Word variations have been searched) 1,026

2. thyroid cancer (Word variations have been searched) 2,019

3. thyroid carcinoma (Word variations have been searched) 969

4. papillary thyroid carcinoma (Word variations have been searched) 337

5. papillary thyroid cancer (Word variations have been searched) 329

6. differentiated thyroid cancer (Word variations have been searched) 598

7. differentiated thyroid carcinoma (Word variations have been searched) 298

8. thyroid tumor (Word variations have been searched) 1,027

9. #1 OR #2 OR #3 OR #4 OR #5 OR #6 OR #7 OR #8 2,411

10. thyroidectomy (Word variations have been searched) 1,737

11. hemithyroidectomy (Word variations have been searched) 70

12. thyroid lobectomy (Word variations have been searched) 85

13. subtotal thyroidectomy (Word variations have been searched) 145

14. #10 OR #11 OR #12 OR #13 1,745

15. thyroid stimulating hormone (Word variations have been searched) 1,362

16. thyroid hormone therapy (Word variations have been searched) 1,940

17. thyroid hormone (Word variations have been searched) 3,339

18. thyroxine (Word variations have been searched) 2,157

19. levothyroxine (Word variations have been searched) 860

20. TSH (Word variations have been searched) 2,602

21. TSH suppression (Word variations have been searched) 300

22. Thyroid Hormone Suppression (Word variations have been searched) 329

23. thyroid stimulating hormone suppression (Word variations have been searched) 188

24. T4 (Word variations have been searched) 6,251

25. thyrotropin (Word variations have been searched) 1,954

26. #15 OR #16 OR #17 OR #18 OR #19 OR #20 OR #21 OR #22 OR #23 OR #24 OR #25 10,345

27. #9 AND #14 AND #26 204
